# Steering Potential for Printing Highly Aligned Discontinuous Fibre Composite Filament

**DOI:** 10.3390/ma16083279

**Published:** 2023-04-21

**Authors:** Narongkorn Krajangsawasdi, Duc H. Nguyen, Ian Hamerton, Benjamin K. S. Woods, Dmitry S. Ivanov, Marco L. Longana

**Affiliations:** Bristol Composites Institute, Department of Aerospace Engineering, School of Civil, Aerospace, and Design Engineering, University of Bristol, Queen’s Building, University Walk, Bristol BS8 1TR, UK; duc.nguyen@bristol.ac.uk (D.H.N.); ian.hamerton@bristol.ac.uk (I.H.); ben.k.s.woods@bristol.ac.uk (B.K.S.W.); dmitry.ivanov@bristol.ac.uk (D.S.I.); marcoluigi.longana@polimi.it (M.L.L.)

**Keywords:** aligned discontinuous fibre, thermoplastic, additive manufacturing, 3D printing, PID control

## Abstract

DcAFF (discontinuous aligned fibre filament) is a novel material for fused filament fabrication (FFF) 3D printing made of highly aligned discontinuous fibres produced using high performance discontinuous fibre (HiPerDiF) technology. It reinforces a thermoplastic matrix to provide high mechanical performance and formability. Accurate printing of DcAFF poses a challenge, especially for complex geometries, because: (i) there is a discrepancy between the path where the filament experiences the adhering pressure from the filleted nozzle and the nozzle path; and (ii) the rasters display poor adhesion to the build platform immediately after deposition, which causes the filament to be dragged when the printing direction changes. This paper explains the implication of these phenomena on steering capabilities and examines the techniques for improving DcAFF printing accuracy. In the first approach, the machine parameters were adjusted to improve the quality of the sharp turning angle without changing the desired path, but this showed insignificant effects in terms of precision improvements. In the second approach, a printing path modification with a compensation algorithm was introduced. The nature of the inaccuracy of the printing at the turning point was studied with a first-order lag relationship. Then the equation to describe the deposition raster inaccuracy was determined. A proportional–integral (PI) controller was added to the equation to calculate the nozzle movement in order to bring the raster back to the desired path. The applied compensation path is shown to give an accuracy improvement in curvilinear printing paths. This is particularly beneficial when printing larger circular diameter curvilinear printed parts. The developed printing approach can be applied with other fibre reinforced filaments to achieve complex geometries.

## 1. Introduction

Fused filament fabrication (FFF), or 3D printing, is a layer-by-layer additive manufacturing technique. FFF builds part by melting solid thermoplastic in a heated nozzle, and then it deposits the molten polymer on a build platform/printing bed or over the previous layer. The polymer cools and forms the desired shape [1,2,3]. It has been shown that the high automation of FFF can reduce manufacturing time, wastage, and cost [4,5]. However, several studies have noted that the current automated manufacturing method of FFF 3D printing creates some geometrical inaccuracy [6,7]. There is an intrinsic inaccuracy associated with 3D printing when the printed features are slightly smaller than the designed part [8,9]. In neat thermoplastic 3D printing, Ai et al. [10] suggested that imperfections in 3D printing can be caused by the shrinkage or crystallization of the polymer. These defects can be very small defects and can be visually undetectable. Furthermore, the commodity thermoplastics used in FFF usually have low mechanical performance, thereby limiting the use of FFF to prototype or secondary (non-load bearing) structures [11,12,13]. This can be addressed by introducing fibre reinforcement to the thermoplastic fibres [11]. Reinforcements can improve the mechanical performance of the thermoplastic [11,14], but the stiff fibres further reduce the printability of thermoplastic, resulting in low dimensional accuracy [15]. This is reflected by the fact that 3D printing of continuous fibre has the lowest ability to perform a circular path or an acute angle compared to neat polymer and short fibre reinforced filaments [9]. The fibre reinforcement can create extra defects associated with the mechanical behaviour of the reinforcement, i.e., twisting and folding of fibre, when printing tight radius curvatures [8,16,17]. Accordingly, the minimum printable radius and angle are limited by the fibre content and the filament diameter. The hardware for fibre printing also influences accuracy. The nozzles used for fibre 3D printing are usually slightly larger than for the pure polymer filaments in order to prevent nozzle clogging and for ease of printing, but this creates gaps between the nozzle wall and the filament. Moreover, before the deposition inside the nozzle, the fibre-reinforced filament, which is smaller than the straight nozzle diameter, can deviate from the vertical axis of the nozzle set to move along the deposition path [18]. The overall printing inaccuracy can cause defects both at the micro-scale, e.g., fibre breakage [16], and at the structural scale, e.g., wrong dimension, which leads to the discarding of the printed part.

Various mathematical models have been constructed to understand fibre printing defects and the deviation from the desired path. For instance, Matsuzaki et al. [8] studied the printing accuracy of continuous carbon fibre impregnated with ABS by printing a circular raster at different radii and found a twisting of fibre during steering, resulting in a smaller radius than the defined path. Based on the experiments, they presented two mathematical models, twisting (where fibre twists and folds back around the curved steering) and path length difference (where the fibres overcome the bed adhesion and move from outside to inside the curvature), to explain the behaviour of the fibre printing and predict the actual printed radius when printing a circular path at different radii with stiff continuous fibre. With the advancement of automation technology, real-time monitoring and machine learning were introduced to 3D printing to detect the printing defect and modified the printing process, e.g., adjusting printing parameters or printing path, so that the printing accuracy and quality can be improved. With neat polymer printing, Delli and Chang [19] used a camera to capture the printing and detect the defects in the semi-finished part. Then, the images were analysed with a supervised machine learning method, support vector machine (SVM), to classify the printing and discard them when the defect was detected. Instead of pausing to take a single shot image, Jin et al. [20] installed a real-time video camera to monitor the printing and then the images were fed to a model, a trained machine learning algorithm, to find the printing defect. If an issue was detected, e.g., over or under extrusion, there is a command to automatically and instantly adjust the printing parameters, e.g., the feed rate and the layer thickness, in the 3D printer controlling unit. This method is faster than human interaction. A similar idea was adapted to continuous fibre printing proposed by Lu et al. [21]. A high-resolution video camera was attached to the 3D printer for real-time defect detection, and then training and supervised deep learning was performed, leading to closed-loop self-adjusting printing parameters to reduce defects, i.e., fibre misalignment and the abrasion between rasters.

**Figure 1:**
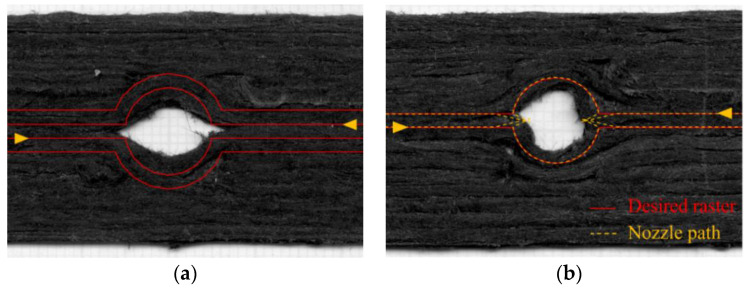
(**a**) Open hole printed part with normal curvilinear printing path; (**b**) open hole printed part with a manual printing path modification by moving the nozzle further inwards (the yellow arrows indicate the printing direction).

In this work, aligned discontinuous carbon fibre preforms produced with high performance discontinuous fibre (HiPerDiF) technology were used as a reinforcement for a FFF thermoplastic filament, now called DcAFF (discontinuous aligned fibre filament). HiPerDiF is a water-based fibre alignment technology to align discontinuous fibres (1–15 mm fibre length) by applying a sudden momentum change to a fibre-water suspension through the impact on the furthermost of two parallel plates [22]. The high level of fibre alignment and the use of short fibres, which are longer than the critical fibre length, leads to high mechanical performance that matches continuous fibre composites while providing better formability [23]. The DcAFF is obtained by reshaping a thin HiPerDiF tape to a circular cross section (0.8 mm diameter) using a purpose-designed machine [24]. When combined with poly(L-lactic acid), or PLA, as the matrix, the DcAFF-PLA mechanical performance is superior to other PLA composite 3D printed materials reported in the literature [24,25]. However, the printability of DcAFF has not been broadly studied. In a previous study on mechanical performance, DcAFF was printed into an open-hole specimen with a defined curvilinear printing path combining straight-line printing with half circles. The printing accuracy is relatively low: it can be seen in Figure 1a that the hole size (from the middle of the top and bottom rasters) is smaller than the desired path (10 mm), and the transition from the linear to the circular path created a deviation from the programmed raster trajectory, giving an eye-shape to the hole. Although a manual additional path modification was attempted by moving the nozzle further inward to reduce the eye shape at the turning corner (Figure 1b), the hole remained asymmetrical (not circular as per design) due to the nozzle dragging the printed raster. This highlights the need for further studies to improve printing geometry accuracy.

This paper aims to explore routes for improving the FFF printing accuracy of DcAFF material. In the first approach, additional printing actions, i.e., the adjustment of the printing parameters, have been coded using a computer numerical control (CNC) programming language. In a second attempt, the initially desired raster has been modified using the instrumentarium of proportional–integral–derivative (PID) control to compensate for the expected discrepancy between the desired and predicted paths. A PI controller was applied to generate a compensated printing path. The results of this modification will be benchmarked against nominal printing for the cases of the corner sections and a 10 mm diameter open-hole sample.

## 2. Materials and Methods

### 2.1. Material

This study used a PLA-based composite 3D printing filament known as DcAFF, which is a 3D printing filament reinforced with aligned discontinuous carbon fibres. The composite material is the combination of a biopolymer (PLA) matrix, supplied by Goodfellow Cambridge Ltd., Huntingdon, England [26], and Toho Tenax 3 mm chopped carbon fibre, 7 μm diameter and coated with water-soluble sizing [27]. A dry-aligned discontinuous fibre preform (approximately 24–26 g/m^2^) produced using the HiPerDiF method was impregnated with a thin PLA tape (62 g/m^2^). The composite tape was reshaped into a circular cross section filament using a bespoke filament-forming machine [24]. The filament has a nominal diameter of 0.8 mm, and the fibre weight content ranges between 20 and 30%.

### 2.2. Printing

**Figure 2:**
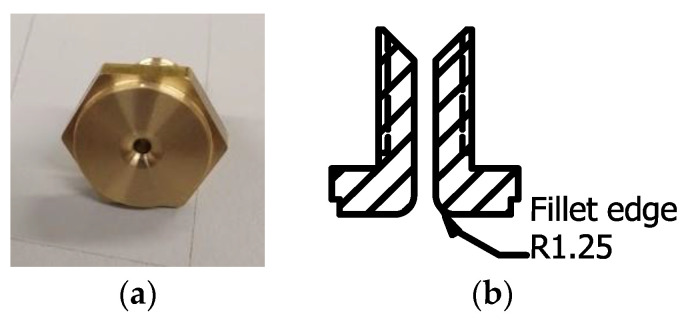
Modified nozzle of 1.4 mm diameter, flat end, and filleted at the outlet: (**a**) the actual nozzle; (**b**) cross section of the side view. Reprinted with permission from [25]. Copyright 2021, Elsevier.

Printing was performed using an Ender3 3D printer with the 1.4 mm diameter modified flat brass nozzle shown in Figure 2. Under normal printing conditions, the printing parameters were: nozzle temperature 210 °C, bed temperature 80 °C, nozzle moving speed and material feed rate 300 mm/min, set nozzle height 0.3 mm, and raster gap 1.6 mm. The parameters were defined according to a previous optimization study [25]. The printing bed was covered with blue masking tape for the 3D printer in order to increase the bed adhesion.

## 3. Result and Discussion

### 3.1. Circular Printing Study

**Figure 3:**
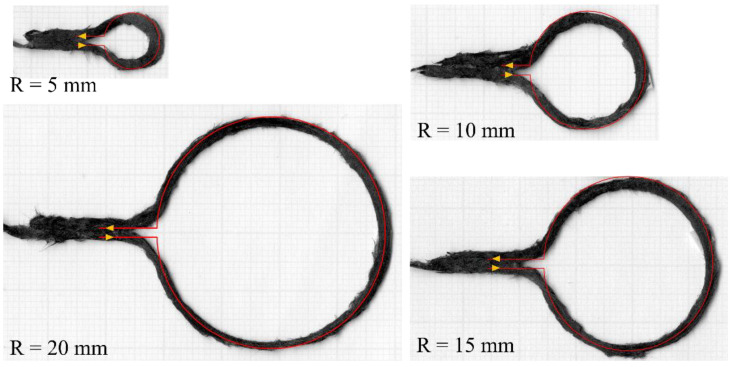
Deposited rasters at different radii from 5 to 20 mm presenting with the desired path (red line), same as the nozzle path, to show the dimension accuracy.

**Figure 4:**
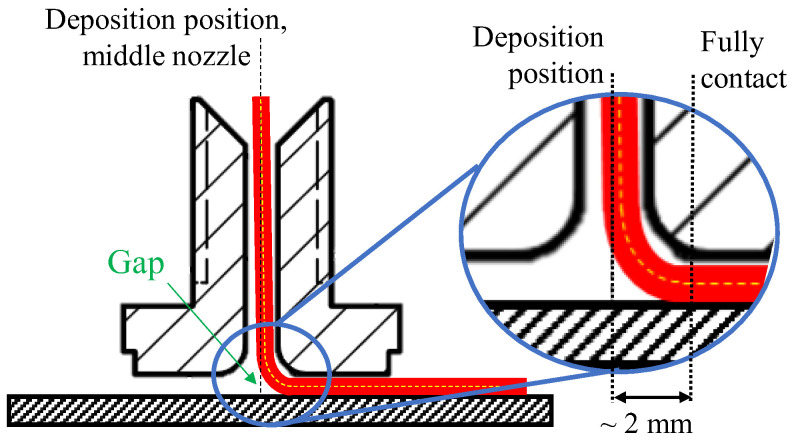
The gap between the filament and the build platform due to the nozzle fillet end creating an offset of the full contact position, and the deviation of filament to the vertical middle line of the nozzle due to the smaller filament diameter than the nozzle diameter.

The dimensional accuracy of the DcAFF filament was investigated by printing circles with different radii, from 5 to 20 mm, with a linear entry and exit. The nozzle path is defined incrementally, in a series of discrete linear steps following the circle contour. Representative results of the printed rasters are shown in Figure 3. It can be seen that the sharp change of path, such as at the entry and exit of the circular part, creates a triangular space between the entering and exiting rasters or fibre bridging between the circular and straight segments. In addition to these artefacts, the circle diameter (the centre of the raster is used for its measurements) is slightly smaller than the defined diameter; the difference ranges from ~4% in the large circular radius (20 mm) to 20% in the small radius (5 mm). The poor dimensional accuracy is caused by the nozzle geometry, the property of the filament, and the adhesive property between the filament and the substrate. The nozzle is filleted, which aims to offer a gentle direction change to the filament being deposited from vertical in the nozzle to horizontal on the build platform without breaking the fibres. Due to the limited stiffness of reinforced filament, this results in a small gap between the deposited material and the build platform, as sketched in Figure 4, which will affect the adhesion of the deposited raster. The point of pressure applied by the nozzle is offset from the centre of the nozzle, which follows the nozzle path. The location of the pressure application changes depending on the angle of the deposition and does not match the position where maximum adhesion needs to be achieved.

The second aspect of the problem is the behaviour of the deposited filament. To hold the filament in place, a sufficient adhesion bond must be formed. This requires longer processing times and higher pressure of the nozzle at the nip point. If the bonding is not fully formed, then bending stresses built in the reinforcements drive the filament from the desired path.

### 3.2. Printing Modification by Tuning Printing Parameters

As highlighted in the previous section, the maximum discrepancy between the desired and printed paths occurs at the point when the straight path turns 90° to the curved path. Hence, this feature can be explored in detail on a simpler corner geometry. This can be simplified into a 90° turning point. This section will, therefore, focus only on sharp turning angles, and describes the first attempt to correct the poor printing at the 90° turning corner without changing the nozzle path. In this case, the nozzle path coincides with the desired geometry. This will study only the modification of the printing parameters to build more adhesion to a specific point, which is expected to reduce the gap created by a 90-degree corner turn. The desired raster and the nozzle path are the same as a 30 mm × 30 mm square with one loop raster. Generally speaking, there are three possible additional printing actions: (i) slow down in the vicinity of the corner to increase adhesion time; (ii) pause the movement at the corner; (iii) increase applied pressure by moving the nozzle downwards. It is possible to create combinations of these three actions in a single run, so the tested cases are (shown in Table 1):

- P1: reduce the print speed from 300 mm/min to 200 mm/min at 5 mm before the corner and accelerate to 300 mm/min at 5 mm after the corner;
- P2: pause at the corner for 1 s before moving forward;
- P3: reduce the nozzle height (stamping) from 0.3 to 0.2 mm at the corner;
- P4: reduce nozzle height to 0.2 mm at 5 mm before the corner and rise back to 0.3 mm at 5 mm after the corner;
- P5: same as P4 plus reducing speed from 300 mm/min at 5 mm before the corner to 200 mm/min at the corner, and rise back to 0.3 mm height at 5 mm after the corner while increasing speed back to 300 mm/min.

**Table 1:** Printing parameter adjustment to tackle the 90° turning corner.

	Speed (mm/min)			Nozzle Height (mm)		
	5 mm Before	At Corner	5 mm After	5 mm Before	At Corner	5 mm After
P1	300	200	300	0.3	0.3	0.3
P2	300	0 for 1 s *	300	0.3	0.3	0.3
P3	300	300	300	0.3	0.3 → 0.2 **	0.3
P4	300	300	300	0.3	0.2	0.3
P5	300	200	300	0.3	0.2	0.3

* Pause for 1 sec at the corner. ** Stamping at the corner (reduce height 0.3 to 0.2 mm).

**Figure 5:**
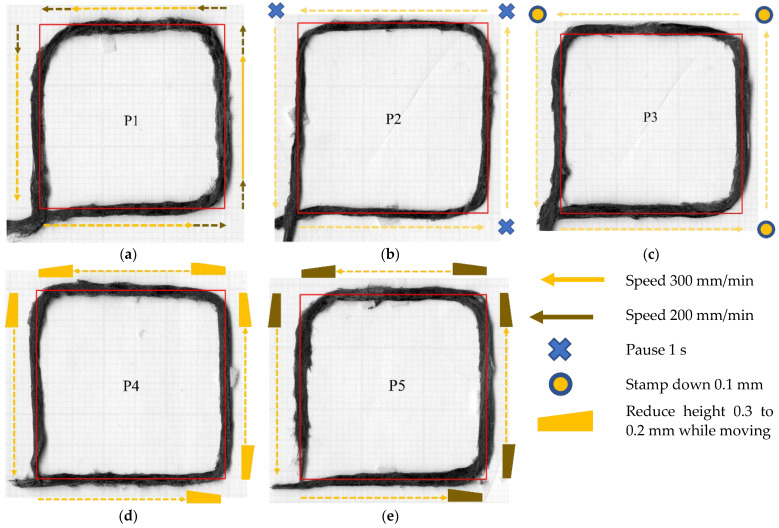
Additional machine actions to increase the printing accuracy following the P1 to P5 described above. (**a**) P1. (**b**) P2. (**c**) P3. (**d**) P4. (**e**) P5.

Figure 5 shows the rasters deposited using the modified actions. It is evident that none of the proposed modifications achieved the required adhesion to eliminate the fibre bridging at the corner. The important contributor to this is the lack of pressure in the centre of the filleted nozzle due to the gap described above (Figure 4). Irrespective of bonding time and applying pressure by the entire nozzle, the pressure at the nip point cannot be sufficiently built. That causes an imperfect bonding of the raster to the build platform at the turning position; the unbonded deposited raster was dragged by the nozzle when it suddenly changed the printing direction. Hence, simple modifications of the printing parameters alone cannot provide good printing accuracy. A different method to reduce the corner gap by adjusting the nozzle path is needed.

### 3.3. Printing Modification by Adding Compensation Coordinates

Section 3.1 has highlighted the challenges of printing along a defined path due to the discrepancy between the centre nozzle position (nozzle path) and the raster–surface contact point. Generally, the “(initially) desired path” is directly converted into the “nozzle path” by the G-code generator and then printed to obtain the “deposited raster”. Owing to the low deposition accuracy of the deposited raster, the nozzle path needs a compensation feature to correct the printing path for any turning angle.

**Figure 6:**
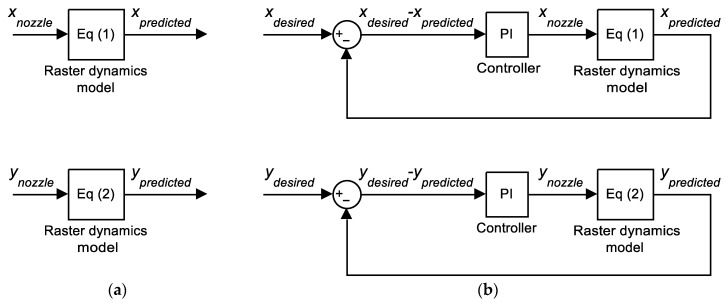
Schematic of the raster dynamics simulation (**a**) without and (**b**) with proportional and integral (PI) compensation.

The first step in building the compensation algorithm is to numerically model the nozzle–raster lag. It is noted that any step change in the nozzle travel direction requires some time for the raster to ‘catch up’ before they both travel in the same direction again. This relationship resembles a first-order lag, which can be approximated by Equations (1) and (2):

(1) $${\dot{x}}_{predicted}=\tau \left(x_{nozzle}-x_{predicted}\right)$$

(2) $${\dot{y}}_{predicted}=\tau \left(y_{nozzle}-y_{predicted}\right)$$

where $x_{nozzle}$ and $y_{nozzle}$ are the x- and y-coordinate of the nozzle centre (in mm). Movement of the nozzle via $x_{nozzle}$ and $y_{nozzle}$ will result in the raster being printed at locations with coordinate $x_{predicted}$ and $y_{predicted}$. Their time derivatives ${\dot{x}}_{predicted}$ and ${\dot{y}}_{predicted}$ represent the component of predicted velocity, and τ is an empirically-derived constant (in s^−1^). The block-diagram representation of this first-order lag is shown in Figure 6a. This simple approximation is only valid when the nozzle velocity is constant, which was kept at 5 mm/s in all experiments.

**Figure 7:**
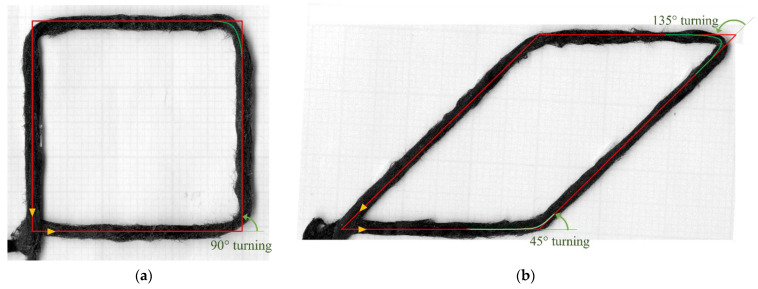
Preliminary shape study (before compensation): (**a**) 90° square; (**b**) parallelogram 45°/135°, green lines show the turning angle before changing printing direction.

**Figure 8:**
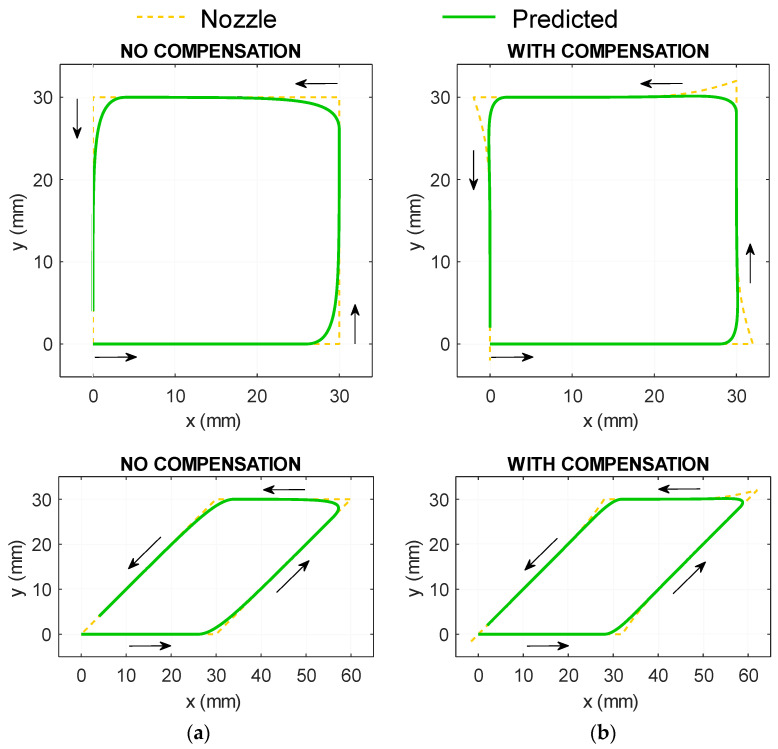
Simulated nozzle path without (**a**) and with (**b**) compensation, starting from (0,0) and printed following the arrows), showing the predicted raster path. The proportional and integral gains used by the PI controllers in (**b**) are *K_P_* = 1.5 and *K_I_* = 2.5.

The value of τ was empirically derived by comparing the predicted movement (Equations (1) and (2)) with the actual deposited paths in printing trials. Since the algorithm should be able to improve the accuracy for any turning angle, these trials are not only performed on the 90° turning case mentioned in the previous section, Figure 7a, but also on other representative turning angles, represented by an acute angle (45° turning) and an obtuse one (135° turning), Figure 7b. Then numerical simulations were conducted with different values of τ until a good match between simulation and experimental results (Figure 7) was achieved. This approach results in τ = 1.25 s^−1^. The simulation result of the nozzle and predicted path based on Equations (1) and (2) using τ = 1.25 s^−1^ is shown in Figure 8a.

With the nozzle–raster relationship identified, the next step is to derive a method by which adjust the nozzle trajectory, so that the raster movement is more closely aligned with the ideal shape. A proportional–integral (PI) controller [28] is deployed to calculate the nozzle path, as shown in the closed feedback loop in Figure 6b. The PI controller then calculates the necessary nozzle movement ($x_{nozzle}$ and $y_{nozzle}$) while minimising the difference between the desired path ($x_{desired}$ and $y_{desired}$) and the predicted path. The mathematical description of this algorithm follows conventional PI controller principles (Equations (3) and (4)):

(3) $$x_{nozzle}=\left(x_{desired}-x_{predicted}\right)K_{P}+K_{I}\int \left(x_{desired}-x_{predicted}\right)dt$$

(4) $$y_{nozzle}=\left(y_{desired}-y_{predicted}\right)K_{P}+K_{I}\int \left(y_{desired}-y_{predicted}\right)dt$$

The coordinates ($x_{nozzle}$ and $y_{nozzle}$) are updated in the iterative process until the desired path is achieved with sufficient accuracy. The two parameters of the PI controller are the proportional gain *K_P_* and integral gain *K_I_*, both of which determine the trajectory of the compensated nozzle movement. The method to determine these gains is based on a similar fitting system, as described in the following section. When the convergence to the right nozzle path is achieved, the compensated nozzle path can be fed to the printer. The examples of compensated paths are shown in Figure 8b.

#### 3.3.1. Tuning the Compensation Algorithm

Experiments have indicated that optimum τ depends on the turning angle, although this value hovers around 1.25 s^−1^ (for the current deposition speed of 5 mm/s). Changing τ, in turn, affects the raster dynamics model, thereby requiring a different set of *K_P_* and *K_I_* to ensure accurate compensation. Identifying the relationship between τ, *K_P_*, and *K_I_* requires an extensive assessment that is beyond the scope of this paper, so some simplification has to be made. It was found that increasing *K_P_* beyond 1.5 provided negligible modification to the compensation path. *K_I_*, on the other hand, showed a significant influence on the compensated path. Therefore, it was decided to fix τ and *K_P_* at 1.25 and 1.5, respectively, while *K_I_* was determined empirically by running different numerical simulations of the compensated algorithm. Such an adjustment provides a pragmatic empirical solution and the demonstration of the compensation algorithm, though, for arbitrary shapes, these dependencies need to be explored with greater detail. These sets of nozzle trajectories were then verified in real experiments. The values of *K_I_* that offered the best result (the print shape is closest to the desired shape) were then chosen.

The compensation algorithm was applied to the following two shapes printed following a spiralling trajectory made of three parallel rasters:

- A 30–60° triangle that represents 90–120–150° turning.
- A parallelogram that represents 45° and 135° turning.

**Figure 9:**
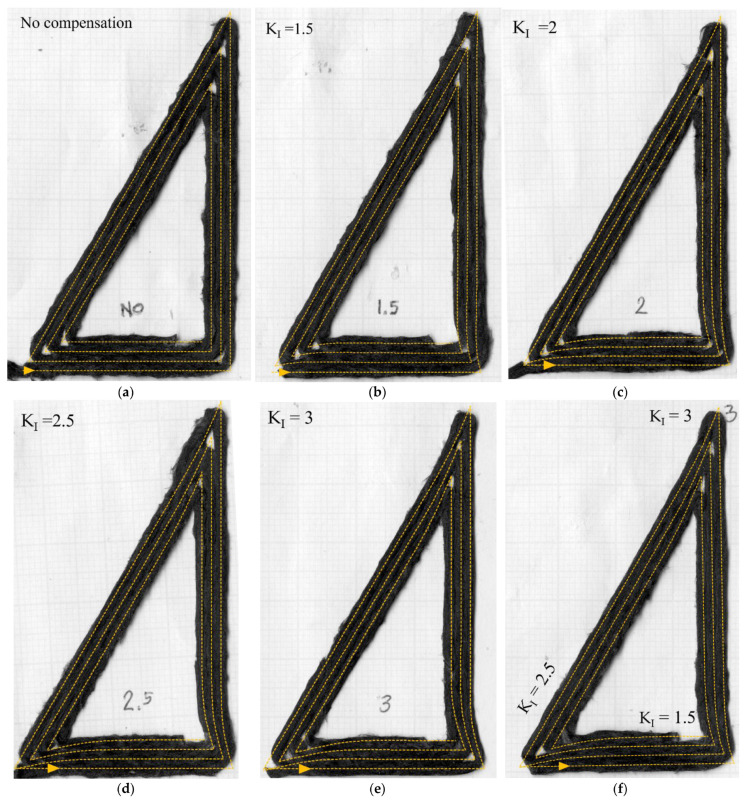
Triangle deposited rasters: (**a**) without compensation; (**b**–**e**) after path compensation with different levels of *K_I_* from 1.5 to 3; and (**f**) with varied *K_I_* (*K_I_* =1.5 at 90°, 2.5 at 120°, 3 at 150° turning angle).

**Figure 10:**
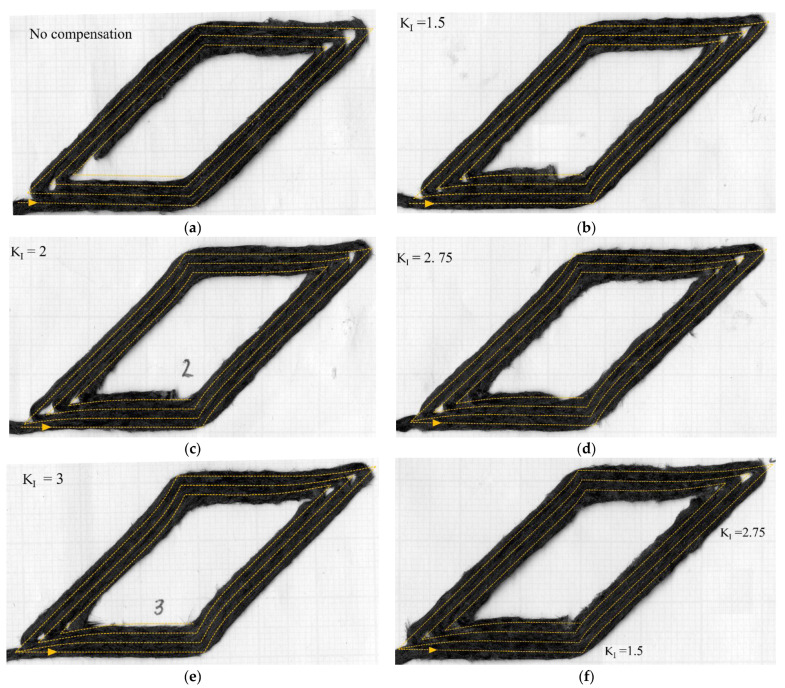
Parallelogram deposited rasters: (**a**) without compensation; (**b**–**e**) after path compensation with different levels of *K_I_* from 1.5 to 3, and (**f**) with varied *K_I_* (*K_I_* =1.5 at 45°, 2.75 at 135° turning angle).

**Figure 11:**
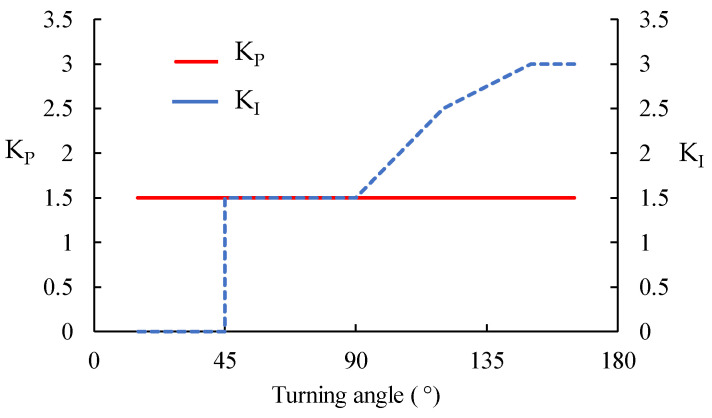
Controller gains, *K_P_* and *K_I_*, that are suitable for specific turning angles according to the experimental result.

Figure 9a and Figure 10a show the spiralling triangle and parallelogram trajectories printed part without compensation. Figure 9b–e and Figure 10b–e are the printed parts with different *K_I_*. The deposited raster is shown with the compensated nozzle path (yellow dotted line). The printing trials show that the magnitude of compensated overshooting is directly related to the *K_I_* variable. Smaller *K_I_* gives smaller compensated overshooting, and vice versa. In Figure 9b, the *K_I_*-1.5 print result performs well with 90° turning, while the higher *K_I_* creates longer overshooting, which generates an extra curvature at the turning point. In Figure 9d–e, the higher *K_I_*, 2.5–3, works well with the large turning angle. The corners are filled-up, especially at the 120° turning (Figure 9e), better than using the low *K_I_*-1.5, Figure 9b, though there is some excess length on the sharp corner at 150° or 135° turning. At the 135° corner (small turning angle of 45°), the *K_I_* 1.5–2 cases (Figure 10b,c) performed well in terms of filling and building up the accurate corners. According to this information, the high *K_I_* values of 2.5–3 are suitable for the high turning angle, and the low *K_I_* of 1.5 is suitable for the low turning angle. The resulting relationship between turning angle and PI gains is shown in Figure 11. A printing test with variable *K_I_* based on the turning angle, Figure 9f and Figure 10f, show that this gives a suitable compensation and a good match between the desired path and the deposited one.

#### 3.3.2. Path Compensation of Curvilinear Printing

**Figure 12:**
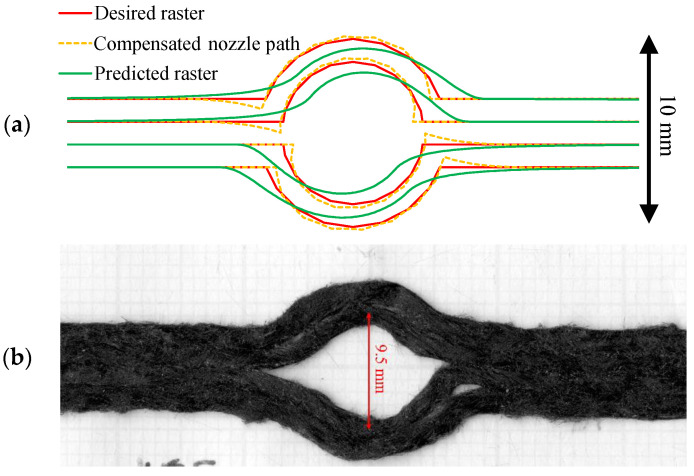
10 mm diameter curvilinear open hole part (D10): (**a**) coordinates of desired raster (red), compensated nozzle path (yellow dotted) and predicted raster (green); (**b**) deposited raster.

According to the open-hole printing accuracy problem mentioned in the introduction, the path compensation from the previous calculation was applied to the open-hole curvilinear to improve the printing quality. The curvilinear desired path was constructed by a series of cartesian coordinates (XY points). In this curvilinear path, the turning angles are always less than 90°, so the most suitable settings are *K_P_*-1.5 and *K_I_*-1.5. In Figure 12, the compensation algorithm is applied to the open-hole 10 mm diameter (D10) desired raster (red) to create the compensated nozzle path (yellow dotted). The compensation algorithm also predicts the printed raster (green), which appears slightly deviated from the centre of the circle. The dimension of the actual deposited raster using the compensation algorithm, shown in Figure 12b, was closer to the desired raster (10 mm diameter curvilinear) than the curvilinear open-hole sample, shown in Figure 1 as the larger circular diameter. However, it is still inaccurate in the hole diameter (middle of each raster), which is quite far off the desired raster. The compensated nozzle path is asymmetric between the entry and exit of the curvature. At the entry corner, the nozzle moved further inwards to the circle centre and dragged the raster in that direction, resulting in a curved deposited raster at the corner similar to the non-compensated path. On the other hand, at the exit corner, the vertical (upwards/downwards) compensated movement of the nozzle can hold the raster at the desired position, generating a sharp turning corner at the transition point. Due to the imperfect curvature shape seen in Figure 12b, it can be implied that the curvature (5 mm radius) is too small, compared to the raster width (1.6 mm) and the filament diameter (0.8 mm), to provide geometrical accuracy. In addition, the length of the fibre (3 mm long) is comparable to the radius of curvature (5 mm), which significantly increases the in-plane bending stiffness of the filament.

**Figure 13:**
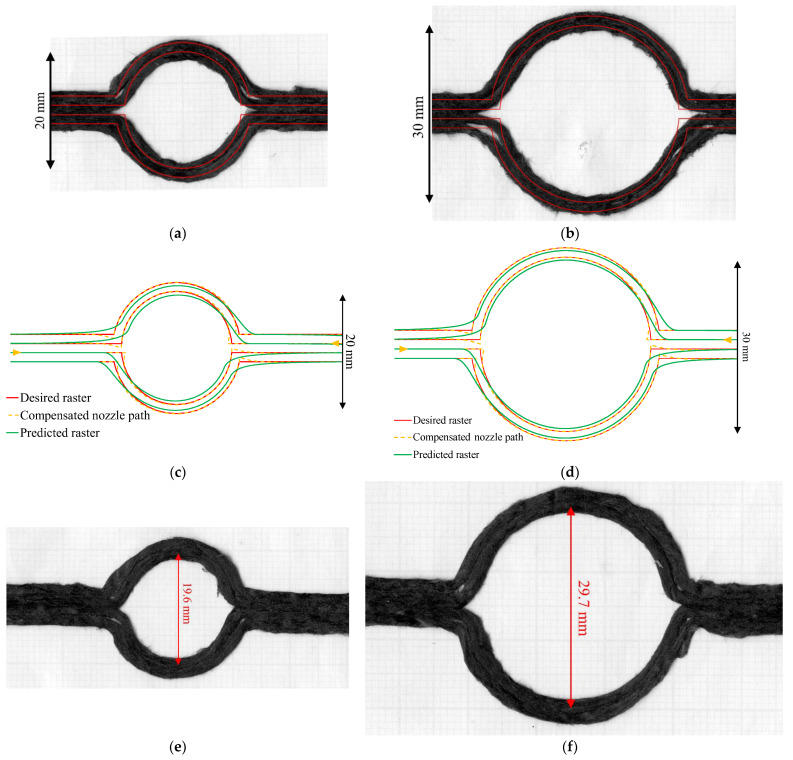
(**a**,**d**) Curvilinear printed parts of 20 and 30 mm diameter without path compensation; (**b**) and (**e**) the path compensation of a 20 and 30 mm diameter circle calculated with the PI controller (yellow dotted) showing the predicted printed path (green) and the desired raster (red); (**c**) and (**f**) curvilinear printed part of 20 mm and 30 mm diameter with the compensation.

The size limit is investigated further using larger diameters than 10 mm, such as 20 mm (D20) and 30 mm (D30). The deposited rasters, D20 and D30, without compensation, are shown in Figure 13a and Figure 13d, respectively. The circle diameters of D20 and D30 are more accurate than the diameter measured on the 10-mm-diameter curvilinear, but the eye-shape issue at the corner is still present. This causes two noticeable gaps at the entry–exit corners that need to be filled with raster.

Using the proposed compensation algorithm, the new nozzle paths for D20 and D30 were generated, as seen in Figure 13b,e. Visually looking at the actual deposited raster in Figure 13c,f, the hole dimensions are almost the correct size, 19.6 and 29.7 mm, respectively, and the eye-shape effect at the corner is reduced. This is more accurate than the uncompensated parts in Figure 13a,d. At the transition, the top and bottom raster become closer to each other in the 20 mm diameter; this is slightly better in the larger diameter (30-mm diameter). This may be caused by the larger curvature size allowing more nozzle travelling time that may create a better bed adhesion. The filling of the second raster is slightly better than with no compensation. The second raster corner is almost fully filled by the overshooting of the second raster.

To quantitatively show the improvement achieved by the algorithm, the compensation of curvilinear printing was further numerically analysed with error calculation between the deposited raster and the desired path using root–mean–square–error (RMS) equation, Equation (5), where $V_{D}$ is the vertical deviation between the desired path to the centre of the deposited raster and *N* is the number of the calculation point. This use of RMS for the evaluation of 3D printing errors was previously suggested by Sugiyama et al. [29] and Reich et al. [30].

(5) $$\mathrm{RMS}=\sqrt{\frac{1}{N}\sum_{i=1}^{n}\left(V_{D}\right)}$$

**Figure 14:**
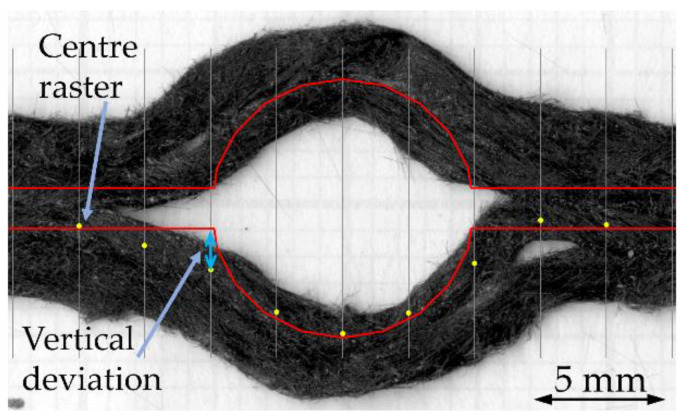
An example of the deviation calculation of the 10 mm compensated curvilinear part showing the calculated (marked) points and the measured vertical deviation.

In our case, the curvilinear deposited rasters were divided horizontally by a 2.5 mm increment from the middle of the circle to 0.5 mm on the linear section at the entry and exit of the curvilinear (another two points before the entry and after the exit of the curvilinear), as can be seen in the example of the 10 mm curvilinear with compensation in Figure 14. Then the centre of the deposited raster at the various positions of the divided curvilinear was marked as a calculation point (*N*). There are 9, 13, and 17 calculation points on the single curvilinear raster for D10, D20, and D30 cases, respectively, according to the size of the part. Using the image processing in ImageJ software, the vertical deviation ($V_{D}$) from the desired raster (red line) to the calculation point on the deposited raster (yellow point) was measured. The deviation at different positions was recorded and converted to root–mean–square values for the path deviation analysis. The RMS vertical deviation values are presented in Table 2. According to the table, the D10 curvilinear shows a minor accuracy improvement after the compensation. On the other hand, there is a strong reduction (by a factor of two) of error in the compensated D20 and D30. When normalised by the diameter of the circle, the RMS of the D20 and D30 cases become similar. These metrics also show that the highest error is characteristic of higher curvatures. This confirms that there is a size limit for this compensation algorithm.

## 4. Conclusions

This study was conducted to improve the printing accuracy of the novel 3D printing composite material, DcAFF, HiPerDiF aligned discontinuous fibre preform and thermoplastic matrix. Previously, the deposited rasters deviated from the defined path due to nozzle design and insufficient bed adhesion. To solve this problem, at first, the machine parameters were adjusted at the turning corner, but this was still insufficient to achieve a sharp turning angle. A printing path compensation algorithm was built with a first-order lag relationship and PI controller, which provides an overshooting segment. This helps the raster to fully attach to the build platform before turning and gives a possibility to steer the DcAFF material in order to achieve a better final geometry. Currently, this compensation scheme is sufficient for rudimentary paths, e.g., turning corners or large radius of curvature (>5 mm). For further development, a real-time monitoring system, i.e., cameras and sensors, can be added to detect the path deviation, and the feedback from those components can be used as a real-time path adjustment to achieve more accurate printing.

## Figures and Tables

**Table 2:** RMS of vertical deviation measuring from the desired path and the centre of the deposited raster.

Printing Diameter	Calculation Points	Compensation		Normalized RMS by Diameter	
		NO	YES	NO	YES
D10	9	0.76	0.73	0.076	0.073
D20	13	0.91	0.45	0.046	0.022
D30	17	1.28	0.64	0.043	0.021

## Data Availability

All data required for reproducibility are provided within the paper.

## References

[1] Ngo T.D., Kashani A., Imbalzano G., Nguyen K.T.Q., Hui D. (2018). Additive manufacturing (3D printing): A review of materials, methods, applications and challenges. Compos. Part B Eng..

[2] Perez A.R.T., Roberson D.A., Wicker R.B. (2014). Fracture surface analysis of 3D-printed tensile specimens of novel ABS-based materials. J. Fail. Anal. Prev..

[3] Gardner J.M., Sauti G., Kim J.-W., Cano R.J., Wincheski R.A., Stelter C.J., Grimsley B.W., Working D.C., Siochi E.J. (2016). Additive Manufacturing of Multifunctional Components using High Density Carbon Nanotube Yarn Filaments.

[4] Wu W., Geng P., Li G., Zhao D., Zhang H., Zhao J. (2015). Influence of layer thickness and raster angle on the mechanical properties of 3D-printed PEEK and a comparative mechanical study between PEEK and ABS. Materials.

[5] Hou Z., Tian X., Zhang J., Li D. (2018). 3D printed continuous fibre reinforced composite corrugated structure. Compos. Struct..

[6] Kumar N., Jain P.K., Tandon P., Pandey P.M. (2018). The effect of process parameters on tensile behavior of 3D printed flexible parts of ethylene vinyl acetate (EVA). J. Manuf. Process..

[7] Geng P., Zhao J., Wu W., Ye W., Wang Y., Wang S., Zhang S. (2019). Effects of extrusion speed and printing speed on the 3D printing stability of extruded PEEK filament. J. Manuf. Process..

[8] Matsuzaki R., Nakamura T., Sugiyama K., Ueda M., Todoroki A., Hirano Y., Yamagata Y. (2018). Effects of set curvature and fiber bundle size on the printed radius of curvature by a continuous carbon fiber composite 3D printer. Addit. Manuf..

[9] Fruleux T., Castro M., Correa D., Wang K., Matsuzaki R., Le Duigou A. (2022). Geometric limitations of 3D printed continuous flax-fiber reinforced biocomposites cellular lattice structures. Compos. Part C Open Access.

[10] Ai J.-R., Peng F., Joo P., Vogt B.D. (2021). Enhanced Dimensional Accuracy of Material Extrusion 3D-Printed Plastics through Filament Architecture. ACS Appl. Polym. Mater..

[11] Krajangsawasdi N., Blok L.G., Hamerton I., Longana M.L., Woods B.K.S., Ivanov D.S. (2021). Fused Deposition Modelling of Fibre Reinforced Polymer Composites: A Parametric Review. J. Compos. Sci..

[12] Tian X., Liu T., Yang C., Wang Q., Li D. (2016). Interface and performance of 3D printed continuous carbon fiber reinforced PLA composites. Compos. Part A Appl. Sci. Manuf..

[13] Sodeifian G., Ghaseminejad S., Yousefi A.A. (2019). Preparation of polypropylene/short glass fiber composite as Fused Deposition Modeling (FDM) filament. Results Phys..

[14] Brenken B., Barocio E., Favaloro A., Kunc V., Pipes R.B. (2018). Fused filament fabrication of fiber-reinforced polymers: A review. Addit. Manuf..

[15] Blok L.G., Longana M.L., Yu H., Woods B.K. (2018). An investigation into 3D printing of fibre reinforced thermoplastic composites. Addit. Manuf..

[16] Shiratori H., Todoroki A., Ueda M., Matsuzaki R., Hirano Y. (2020). Mechanism of folding a fiber bundle in the curved section of 3D printed carbon fiber reinforced plastics. Adv. Compos. Mater..

[17] Yamawaki M., Kouno Y. (2018). Fabrication and mechanical characterization of continuous carbon fiber-reinforced thermoplastic using a preform by three-dimensional printing and via hot-press molding. Adv. Compos. Mater..

[18] Tu Y., Tan Y., Zhang F., Zhang J., Ma G. (2019). Shearing algorithm and device for the continuous carbon fiber 3D printing. J. Adv. Mech. Des. Syst. Manuf..

[19] Delli U., Chang S. (2018). Automated process monitoring in 3D printing using supervised machine learning. Procedia Manuf..

[20] Jin Z., Zhang Z., Gu G.X. (2019). Autonomous in-situ correction of fused deposition modeling printers using computer vision and deep learning. Manuf. Lett..

[21] Lu L., Hou J., Yuan S., Yao X., Li Y., Zhu J. (2023). Deep learning-assisted real-time defect detection and closed-loop adjustment for additive manufacturing of continuous fiber-reinforced polymer composites. Robot. Comput. Integr. Manuf..

[22] Yu H., Potter K.D., Wisnom M.R. (2014). A novel manufacturing method for aligned discontinuous fibre composites (High Performance-Discontinuous Fibre method). Compos. Part A Appl. Sci. Manuf..

[23] Such M., Ward C., Potter K. (2014). Aligned discontinuous fibre composites: A short history. J. Multifunct. Compos..

[24] Krajangsawasdi N., Woods B.K.S., Hamerton I., Ivanov D.S., Longana M.L. Highly Aligned Discontinuous Fibre Composite Filaments for Fused Deposition Modelling: Open-Hole Case Study. Proceedings of the Composites Meet Sustainability—Proceedings of the 20th European Conference on Composite Materials, ECCM20.

[25] Krajangsawasdi N., Longana M.L., Hamerton I., Woods B.K., Ivanov D.S. (2021). Batch production and fused filament fabrication of highly aligned discontinuous fibre thermoplastic filaments. Addit. Manuf..

[26] Goodfellow Poly L lactic acid-Biopolymer-Film. https://www.goodfellow.com/uk/en-gb/displayitemdetails/p/me33-fm-000150/poly-l-lactic-acid-biopolymer-film.

[27] Teijin Carbon. Tenax^®^Short Fiber Product Data Sheet Chopped Fiber with Thermoplastic Sizing. https://www.teijincarbon.com/products/tenaxr-carbon-fiber/tenaxr-short-fibers.

[28] Åström K.J., Hägglund T. (2001). The future of PID control. Control Eng. Pract..

[29] Sugiyama K., Matsuzaki R., Ueda M., Todoroki A., Hirano Y. (2018). 3D printing of composite sandwich structures using continuous carbon fiber and fiber tension. Compos. Part A Appl. Sci. Manuf..

[30] Reich S., Berndt S., Kühne C., Herstell H. (2022). Accuracy of 3D-Printed Occlusal Devices of Different Volumes Using a Digital Light Processing Printer. Appl. Sci..

